# The co-receptor signaling model of HIV-1 pathogenesis in peripheral CD4 T cells

**DOI:** 10.1186/1742-4690-6-41

**Published:** 2009-05-01

**Authors:** Yuntao Wu

**Affiliations:** 1 Department of Molecular and Microbiology, George Mason University, Manassas, VA 20110, USA

## Abstract

HIV-mediated CD4 depletion is the hallmark of AIDS and is the most reliable predictor of disease progression. While HIV replication is associated with CD4 depletion in general, plasma viremia by itself predicts the rate of CD4 loss only minimally in untreated patients. To resolve this paradox, I hypothesize the existence of a subpopulation of R5_X4-signaling _viruses. I also suggest that the gradual evolution and emergence of this subpopulation are largely responsible for the slow depletion of peripheral CD4 T cells.

## Background

**Figure 1:**
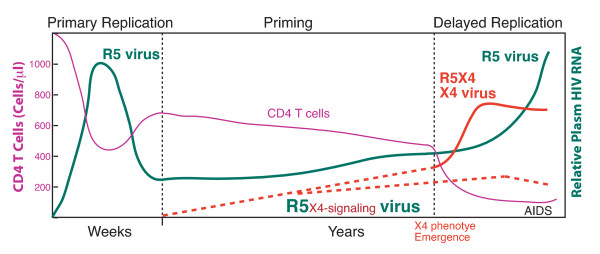
**The co-receptor signaling model of HIV pathogenesis in peripheral CD4 T cells**. In this model, I hypothesize that the emergence of the R5_X4-signaling _viruses (Red dotted lines) is responsible for the slow depletion of peripheral CD4 T cells.

The human immunodeficiency virus (HIV) infects CD4 T cells and causes CD4 depletion which leads to the development of AIDS. In the spectrum of clinical signs associated with HIV infection, CD4 depletion is a hallmark and is one of the most powerful predictors of disease progression. On the other hand, the level of viral replication, as reflected by plasma viral RNA load, has also been suggested to directly predict progression to AIDS and death [1]. Nevertheless, the relationship between plasma viremia and CD4 depletion rate has been a subject of debate [2]. While it is certain that a strong correlation between viral load and CD4 depletion exists when plasma viremia is grouped into different categories (*e.g. *< 500 copies/ml, 501–3000 copies/ml, >30,000 copies/ml *etc.*) [1,3], at the individual level, the presenting viral load poorly predicts the rate of CD4 depletion in untreated patients [2,4]. To resolve this paradox, here I propose a new hypothesis from a co-receptor signaling perspective based on our recent studies [5]. As shown in Figure 1, I hypothesize that HIV-1 gp120-CXCR4 signaling plays a major role in the gradual depletion of peripheral CD4 T cells during chronic HIV infection.

In this model, I separate the disease course into three phases: (1) primary HIV replication, (2) priming, and (3) delayed HIV replication. The primary phase largely involves the efficient replication of CCR5-utilizing, M-tropic viruses such as those replicating in the GI tract [6]. In the second phase, with immune suppression or the consumption of most of the available CCR5 target T cells, viral replication is reduced to a low level. This low-level ongoing viral replication serves as a reservoir that supplies viral mutants to prime the immune system for new target cells. Early on in the priming phase, limited mutations such as one or two amino acid changes in the V3 loop of the viral envelope may give rise to the first CXCR4-priming virus. These small numbers of early viruses may still use CCR5 for entry and replication but can engage CXCR4. This CXCR4 binding may not permit viral entry since successful fusion and entry often require more than two mutations or even mutations outside of the V3 loop [7]. Other virological obstacles may also play a role in preventing the quick emergence of viruses with the X4 phenotype [8]. Nevertheless, these early CXCR4-priming viruses (R5_X4-signaling _viruses) can trigger signal transduction in CXCR4 cells without actually infecting and replicating in these cells. On the other hand, aberrant CXCR4 signaling may mediate CD4 T cell dysfunction and contribute to chronic immune activation, gradually shifting these otherwise restrictive cells towards the direction of permissiveness. With continued engagement of the CXCR4 receptor, the priming event may eventually lead to the emergence of the X4 viral phenotype and its viral replication in some patients. The newly emerged CXCR4-utilizing, T-tropic viruses would then find a large pool of targets and initiate a new phase of viral replication, the third delayed replication phase, which could result in rapid CD4 depletion and fast progression to AIDS [9]. In some patients, the full X4 phenotype may never arise, but the X4 priming could remain an ongoing process that would provoke slow CD4 depletion and disease progression.

The central tenets of this new signaling model are the hypothetical existence of the R5_X4-signaling _viruses during chronic infection and the direct association of these viruses with CD4 depletion. The R5_X4-signaling _viruses are predicted to be a minority during the chronic phase with no strong replication or selection advantage over other R5 viruses [10], largely because of the continuous use of CCR5-positive cells for replication. Moreover, the signaling and depletion of CD4 T cells by the R5_X4-signaling _mutants are likely to be loosely correlated with the overall predominance of R5 viruses which are less pathogenic to peripheral CD4 T cells in general. Nevertheless, the emergence of the R5_X4-signaling _viruses does depend on the pool of R5 viruses; thus, while HIV-1 replication is overall associated with CD4 depletion [1], the use of total plasma viral RNA load, a measurement of mostly R5 viruses, is a poor predictor of the slow CD4 loss in patients [2]. The very existence of the hypothetical R5_X4-signaling _subpopulation that can directly cause CD4 loss would be a reasonable explanation for the observed paradoxical relationship between total viral load and CD4 depletion [1,2].

## Discussion

### The different T cell targets of M-tropic and T-tropic viruses

The natural course of HIV infection almost always starts with the robust replication of the CCR5-ultilizing M-tropic viruses [6,11,12]. The R5 viruses can quickly infect, replicate and kill a large number of target cells such as the active memory CD4 T cells present in the GI tract [6,11,12]. This early process occurs in both HIV-1 infection of humans [6,12] and in the pathogenic and non-pathogenic SIV infection of monkeys [11], and can result in lasting pathogenic insults [13] to or non-pathogenic effects [14,15] on the immune system. With the onset of the asymptomatic phase following the acute infection, viral diversification occurs. In about 50% of infected patients (mostly subtype-B infection), there is a viral switch in the co-receptor usage, from CCR5 to CXCR4, at late stages of disease. This switch correlates with faster CD4 depletion and more rapid disease progression towards AIDS [9,16-19]. The late emergence of the CXCR4-utilizing viruses may be a reflection of the restrictive nature of the X4 viral target cells. In the human immune system, a majority of CD4 T cells in the peripheral blood are CXCR4-positive, resting CD4 T cells. These cells pose numerous restrictions that the virus has to overcome to replicate. Firstly, the viral envelope has to be mutated to engage the CXCR4 receptor [20], and the mutations have to accumulate to a sufficient degree to permit successful viral entry [7,8]. Secondly, the virus has to modulate the immune system, either by inducing cytokines [21-23] or facilitating transient immune activation to permit viral integration [24-29]. Even with successful integration, the virus has to induce or rely on chronic immune activation to maintain stable gene expression and viral production [22,23,30]. Recently, we demonstrated that the static cytoskeletal actin in resting CD4 T cells is also a barrier for viral intracellular migration [5]. To overcome this restriction, the virus has to rely on signal transduction via the viral envelope binding to CXCR4, which triggers the activation of an actin depolymerization factor cofilin in resting T cells. This cofilin activation increases cortical actin treadmilling and actin dynamics, permitting viral migration across the cortical actin barrier [5].

Given the critical role that CXCR4 signaling plays in HIV infection of peripheral CD4 T cells, it is possible that HIV-mediated aberrant signalling through CXCR4 may contribute to viral pathogenesis in these cells. It has long been recognized that the residual CD4 cells in HIV-infected subjects have multiple functional abnormalities such as anergy [31,32], loss of T helper function [33], and abnormal T cell homing and migration [34,35], all of which result from the bystander effect [36]. These T cell abnormalities suggest that although they are not directly infected, these residual CD4 T cells may have been engaged by viruses or viral factors, and their signaling responses to environmental stimuli have been profoundly altered.

### Supporting evidence from bioinformatics studies of the evolution of the HIV envelope protein

In contrast to the R5 viruses, the capacity of the late-emerging X4 viruses to cause rapid CD4 depletion clearly demonstrates the pathogenic importance of the CXCR4-engaging viruses [9,16-19]. Interestingly, by using bioinformatics approaches such as neural networks [37], PSSM [38,39] or 11/25 genotype [39-43], the potential of the R5 virus to switch to the more pathogenic X4 virus can be predicted based on the charged residues within the V3 loop, particularly at the 11 and 25 positions of V3. Remarkably, even though approximately 50% of patients do not actually acquire the X4 phenotype ever in their disease, the V3 genotypes were found to be associated with more rapid CD4 depletion and faster disease progression [44]. The predictive value of the X4 genotypes for CD4 depletion presumably hinges upon the occurrence of the X4 phenotype. Yet, it is very possible that these X4 genotypes may reflect the actual capacity of the viral envelopes to engage and signal through CXCR4. Therefore, the direct correlation of CD4 depletion with the X4 genotypes in the absence of the X4 phenotype is a strong indication of the possible existence of the R5_X4-signaling _viruses. As a matter of fact, a recent study using massive pyrosequencing of the V3 loop has found that clusters of the R5 proviral genomes harboured in patients' monocytes carry mutations with the X4 genotypes [45]. Similar R5 genotypic evolution was also observed even in patients maintaining exclusively the R5 viruses [46]. In the absence of the R5-to-X4 phenotypic switch, the R5 phenotype does evolve with disease progression in properties such as a decreasing sensitivity to the neutralization by CC chemokines [47] and an increasing capacity for direct and DC-SIGN-mediated trans-infection of T cells [46]. In addition, it has also been shown that in the peripheral blood mononuclear cells of infected patients, different sub-populations of infected cells co-exist, and some of these cells, such as infected monocytes and memory T cells, have a slow decay rate [48]. These cells may serve as the seeds for the development of the R5_X4-signaling _phenotype.

### The balance between gp120 priming T cells and triggering apoptosis

In addition to transducing signals to promote HIV infection [5,49-51], HIV envelope binding to the chemokine co-receptors has also been suggested to trigger apoptosis of CD4 T cells [52-56]. Even before the identification of the chemokine co-receptors, gp120 was proposed to trigger activation-dependent T cell apoptosis through the CD4 receptor [57-59]. This suggestion was based on a similar mechanism observed in the activation of murine lymphocytes in which pre-stimulation of the CD4 receptor triggered apoptosis when the cells were also activated through the T cell receptor [60]. It appears that engagement of the CD4 receptor alone, either by the R5 or X4 viruses, may not be sufficient to trigger apoptosis; CD4 signaling promotes apoptosis largely in the presence of signals that also activate CD4 T cells [57-59]. The R5-viruses may induce apoptosis through CCR5 in active memory CD4 T cells [61]. The majority of peripheral resting CD4 T cells, however, have either no CCR5 or low levels of CCR5 receptor. It is possible that the apoptotic process in resting CD4 T cells is triggered by the X4 viruses or X4-signaling viruses by binding and signaling through CXCR4.

HIV envelope-mediated apoptosis has been implicated to contribute to the depletion of either infected or uninfected CD4 T cells [57]. Nevertheless, from a purely virological point of view, triggering apoptosis, especially at the earliest time of infection, is a misfortune and is something that a virus should always avoid. For example, even the fast replicating, extremely cytolytic viruses such as baculovirus encode anti-apoptotic proteins to avoid triggering apoptosis at an early time [62]. In HIV infection, latently infected resting CD4 T cells, with a half life as long as 3 to 4 years [63], were frequently detected to persist in patients [64-66]. In addition, it has also been shown that in contrast to triggering apoptosis, the HIV-1 envelope can induce productive viral replication from the resting CD4 T cells of HIV-infected patients [51]. Therefore, it is possible that even though the HIV envelope triggers apoptosis of CD4 T cells, this may not frequently occur until the X4 signaling viral population reaches a significant level. In other words, the balance between CXCR4 priming and CXCR4 triggering apoptosis is probably regulated by signal strength; apoptosis would require higher viral dosages. In the HIV disease course, initially, low levels of X4 signaling viruses may prime CD4 T cells for infection, whereas at a late stage when levels of X4 signaling viruses are high especially with the emergence of the X4 phenotype, triggering apoptosis may be more common and may directly contribute to CD4 depletion.

It has also been suggested that HIV-infected cells downregulate PD-1, whereas uninfected bystander cells do not [67]. PD-1 downregulation prevents cells from early apoptosis. Presumably, this mechanism would enrich HIV+ CD4 T cells, facilitating the amplification of X4 viruses. Nevertheless, this mechanism probably would not be in play until the late emergence of the X4 phenotype.

### Basic characteristics associated with the hypothetic R5_X4-signaling _viruses

In the chemokine co-receptor signaling model, the hypothetic R5_X4-signaling _viruses are proposed to be responsible for the slow depletion of peripheral CD4 T cells. Experimental demonstration of such R5_X4-signaling _viruses requires the establishment of certain basic criteria. Firstly, the R5_X4-signaling _viruses are phenotypically R5 viruses. They should enter CD4^+^CCR5^+ ^but not CD4^+^CXCR4^+ ^indicator cells in co-receptor tropism assays. These viruses should also demonstrate susceptibility to antagonists specific for CCR5. Secondly, the envelope protein from the R5_X4-signaling _viruses should be able to bind to CXCR4 in non-cell-based *in vitro *binding assays; this interaction can be competitively inhibited by a CXCR4 antagonist. The ability to interact with CXCR4 does not equate with the capability to trigger fusion, which requires the involvement of other regions in addition to the V3 loop of gp120. There are likely varying degrees of affinity for CXC4 among the R5_X4-signaling _viruses. Thirdly, the R5_X4-signaling_viruses should demonstrate the ability to trigger signal transduction through CXCR4 in resting CD4 T cells. The signaling may also be shut down by a CXCR4 antagonist. The issue is complex because CXCR4 signaling is known to be diverse and can activate an array of downstream targets such as Pyk2 [68], PI3K, Akt [69,70], Erk-1/2 [70], and cofilin [5]. It is expected that not every one of these targets is directly involved in HIV infection and pathogenesis. In addition, there are also dosage and affinity-dependent differences in activating specific pathways. For example, at low dosages, SDF-1 binding to CXCR4 attracts CD4 T cells, whereas at high dosages, the same binding does the opposite to repel CD4 cells [35,71]. Therefore, the critical issue becomes what downstream target should be used as a readout for measuring CXCR4 signaling at a defined viral dosage. Currently, we propose coflin as a final readout for measuring CXCR4 signaling in CD4 T cells because we have demonstrated that it is a direct downstream target of gp120-CXCR4 interaction, and its activation facilitates viral infection. Nevertheless, clinical studies are required to determine whether activation of cofilin or any other CXCR4 downstream target is directly associated with CD4 depletion and HIV disease progression. Establishment of this relationship is an essential step to experimentally identify the R5_X4-signaling_subpopulation.

## Conclusion

The co-receptor signaling model implies that the HIV envelope plays a major role in the slow depletion of peripheral CD4 T cells. Although HIV directly infects only a very small percentage of peripheral CD4 T cells (0.2–16.4 HIV-latently infected cells per 10^6 ^resting CD4 T cells [64]), the ability of the viral envelope to alter T cell function through signal transduction should not be underestimated. This hypothesis highlights the need for a thorough examination of the signaling properties of HIV quasispecies in patients. I also speculate that these R5_X4-signaling _viruses may cause cofilin activation in resting CD4 T cells as suggested in our recent studies [5,72]. Conceivably, in comparison with the use of plasma viral load as a readout, cofilin activation would be a more direct reflection of CD4 dysfunction and may serve as an early marker for predicting CD4 depletion.

## Abbreviations

M-tropic: Macrophage Tropic; T-tropic: T cell Tropic; R5: CCR5; X4: CXCR4; GI: Gastrointestinal

## Competing interests

The author declares that he has no competing interests.
